# The Role of Macrophages in the Infarcted Myocardium: Orchestrators of ECM Remodeling

**DOI:** 10.3389/fcvm.2019.00101

**Published:** 2019-07-31

**Authors:** Sinead A. O'Rourke, Aisling Dunne, Michael G. Monaghan

**Affiliations:** ^1^Department of Mechanical and Manufacturing Engineering, Trinity College Dublin, Dublin, Ireland; ^2^School of Biochemistry & Immunology and School of Medicine, Trinity Biomedical Science Institute, Trinity College Dublin, Dublin, Ireland; ^3^Trinity Centre for Bioengineering, Trinity Biomedical Science Institute, Trinity College Dublin, Dublin, Ireland; ^4^Advanced Materials for BioEngineering Research (AMBER) Centre, Trinity College Dublin and Royal College of Surgeons in Ireland, Dublin, Ireland

**Keywords:** macrophages, myocardial infarction, ECM, fibrosis, inflammation, wound healing, immunomodulation, macrophages (M1/M2)

## Abstract

Myocardial infarction is the most common form of acute cardiac injury attributing to heart failure. While there have been significant advances in current therapies, mortality and morbidity remain high. Emphasis on inflammation and extracellular matrix remodeling as key pathological factors has brought to light new potential therapeutic targets including macrophages which are central players in the inflammatory response following myocardial infarction. Blood derived and tissue resident macrophages exhibit both a pro- and anti-inflammatory phenotype, essential for removing injured tissue and facilitating repair, respectively. Sustained activation of pro-inflammatory macrophages evokes extensive remodeling of cardiac tissue through secretion of matrix proteases and activation of myofibroblasts. As the heart continues to employ methods of remodeling and repair, a destructive cycle prevails ultimately leading to deterioration of cardiac function and heart failure. This review summarizes not only the traditionally accepted role of macrophages in the heart but also recent advances that have deepened our understanding and appreciation of this dynamic cell. We discuss the role of macrophages in normal and maladaptive matrix remodeling, as well as studies to date which have aimed to target the inflammatory response in combatting excessive matrix deposition and subsequent heart failure.

## Introduction

Heart failure is a global pandemic, accounting for 31% of deaths worldwide (1). Health expenditures associated with heart failure are substantial, and expected to increase dramatically with an aging population (2). Myocardial infarction (MI) is the most common form of acute cardiac injury attributing to heart failure, and while there have been significant advances in therapies, mortality and morbidity remain high. Our understanding of MI has evolved in recent years with inflammation driven by macrophages now recognized as playing a key pathological role in the progression of tissue remodeling and fibrosis which, in turn, limits cardiac function. A greater appreciation of the role of the inflammatory response and the interaction between macrophages and the extracellular matrix (ECM) is required in order to provide greater insight into tissue remodeling and disease progression within the myocardium, as well as revealing therapeutic targets for the treatment of heart failure. In this review we will discuss the importance and role of macrophages in the healthy and infarcted myocardium, and how these innate immune cells contribute toward ECM remodeling and fibrosis.

## Multicellularity of the Heart

The myocardium is a multicellular complex tissue comprised of a range of distinct cell-types. Cardiomyocytes (CMs) constitute approximately one third of resident myocardial cells by number (3), with the remaining two thirds referred to as non-excitable cells (non-CMs), such as fibroblasts, smooth muscle cells, endothelial cells, autonomic motor neurons, and immune cells, such as mast cells and macrophages (4). While CMs possess inherent conduction capabilities which mediate the characteristic contractile forces of the heart, non-CMs are responsible for matrix deposition, vascularization and autonomic regulation (5). CMs and non-CMs communicate via biochemical signaling through cytokine and growth factor secretion (5, 6). Such signals arise, for example, during development and regulation and include the release of vascular endothelial growth factor (VEGF) which activates endothelial cells to initiate angiogenesis (7), or in response to trauma or injury, where signaling is mediated by pro-inflammatory cytokines such as tumor necrosis factor alpha (TNFα) (8, 9). Numerous networks also exist between non-CMs, such as fibroblasts and macrophages, working in tangent to maintain the structural integrity of the heart.

Fibroblasts are traditionally defined as cells of mesenchymal origin, arising from bone marrow derived cells known as fibrocytes (10, 11). Cardiac fibroblasts produce the necessary components for the construction of the ECM in order to maintain the integrity of the myocardium (10). As a result, fibroblasts have been highlighted as key mediators of both normal cardiac function and the remodeling response to injury (6, 10, 12). In addition to producing components of the ECM, fibroblasts are also observed to secrete regulatory proteins and matrix metalloproteinases as well as their corresponding inhibitors, tissue inhibitors of metalloproteinases, thus maintaining a well-controlled balance for ECM homeostasis (13).

## Macrophages—Key Drivers of the Innate Immune Response

Macrophages (and their precursors, monocytes) are key mediators of the innate immune response involved in the recognition, phagocytosis and elimination of pathogens. They exist as both circulating and tissue resident cells within the body and have the ability to change their function and phenotype based on environmental cues (14). While they exist as a heterogenous population they can be broadly classified as M1 or M2 macrophages (15). M1 macrophages are traditionally associated with a pro-inflammatory response, and are referred to as classically activated macrophages, induced by IFNγ, LPS, and TNFα. When stimulated, M1 macrophages secrete high levels of pro-inflammatory cytokines IL-12, IL-23, IL-1, and IL-6 (16). M2 macrophages, or “alternatively activated” macrophages exhibit an anti-inflammatory, pro-regenerative phenotype largely due to their ability to secrete high levels of anti-inflammatory cytokines including IL-10 and growth factors, such as VEGF as well as matrix metalloproteinases (MMPs) (16). In murine models, M1 and M2 macrophages are distinguished from another through the expression of the inflammatory monocyte marker Ly6C. Ly6C^high^ monocytes are preferentially recruited to sites of inflammation and exhibit an M1 pro-inflammatory phenotype while Ly6C^low^ monocytes represent the non-classical population and differentiate into M2 macrophages to promote tissue healing and angiogenesis (17).

While the M1/M2 paradigm proves useful as a preliminary introduction to these innate immune cells, the full story is not as black and white. The macrophage phenotype exhibits more plasticity than historically assumed, and M1/M2 classification merely represents two extremes of a continuum of activated states. For example, macrophages treated with the pathogen associated molecule, lipopolysaccharide (LPS), exhibit a reduced phagocytic capacity compared to macrophages treated with the endogenous cytokine; IFNγ. While both produce pro-inflammatory mediators, LPS polarized macrophages are now referred to as M1b macrophages, whereas IFNγ polarized macrophages are referred to as M1a macrophages (16, 18).

Distinct M2 macrophage subsets also exist. For example, M2a macrophages are induced by IL-4 and IL-13 and have a pre-dominantly anti-inflammatory phenotype, secreting high levels of IL-10 and IL-1 receptor antagonist as a means of dampening the inflammatory response. M2b macrophages, on the other hand, exhibit both pro- and anti-inflammatory responses, producing IL-1β, TNFα, and IL-6 as well as IL-10 in response to LPS stimulation. M2c macrophages are induced by IL-10 and secrete high levels of transforming growth factor beta 1 (TGFβ1), and glucocorticoids. They assume a regenerative, pro-healing phenotype and play a major role in promoting tissue repair and silencing the inflammatory response. These cells also play a significant role in matrix deposition (15). More recently, M2d and M2f phenotypes have been characterized (19, 20). M2d macrophages are activated by Toll-like receptor agonists and adenosine A2a receptor agonists. In response, these cells secrete high levels of VEGF and IL-10, and in turn downregulate TNFα and IL-12 production (19). M2f cells are induced by efferocytosis which involves the removal of apoptotic cells by macrophages. This process is similar to phagocytosis, however, it involves distinct receptors and signaling pathways and results in the secretion of high levels of TGFβ1, prostaglandin E2 and platelet activating factor, all of which are known to inhibit LPS-induced pro-inflammatory cytokine production (21).

## Tissue Resident Macrophages

Tissue resident macrophages exist at various sites throughout the body and can include microglia in the brain and Kuppfer cells in the liver (22). The heart, being no exception; contains its own resident macrophages which possess a specific role in the regulation of cardiac function (23). The distinction between tissue residing cardiac macrophages and circulating monocyte-derived macrophages has become a considerable area of focus in recent years (24). While long established tissue resident cells appear to facilitate coronary development and tissue homeostasis, it appears that monocyte-derived infiltrating cells have a predominant role in tissue injury and destruction. This highlights that macrophages, whether circulating or permanently residing, originate from diverse lineages, and as a result have different functions.

## CCR2^+^ and CCR2^−^ Tissue Resident Macrophages

Gene mapping of cardiac resident macrophages reveals two distinct lineages arising at the embryonic stage and post-natal stage (25). Developmental studies of early cell migration in murine models affirms this, with the earliest cardiac resident macrophages derived from an erythromyeloid progenitor in the yolk sac (26). These progenitor macrophages migrate out of the yolk-sac either directly to the developing myocardium or else to the fetal liver, where they progress to hemopoietic stem cells and eventually cardiac tissue-resident macrophages (26). Post-natally, monocyte-derived macrophages can also migrate to the myocardium to become tissue resident macrophages (27). These embryonic and post-natal resident cells can be distinguished from one another based on expression of the chemokine receptor, Chemokine Receptor Type 2 (CCR2) (25). This receptor and its corresponding ligand, chemokine ligand 2 (CCL2), also known as monocyte chemoattractant protein 1 (MCP-1), play an important role in monocyte/macrophage migration. Studies have demonstrated that CCR2^+^ cardiac resident macrophages are derived from monocytes while CCR2^−^ macrophages originate from the embryonic developmental stage (25, 28). Furthermore, CCR2^−^ macrophages undergo local proliferation in order to replenish their population whereas CCR2^+^ macrophages are repopulated by monocyte-derived macrophages extravasating into the myocardium (25). Both CCR2^+^ and CCR2^−^ cell populations orchestrate diverse responses following traumatic events, such as MI. CCR2^+^ cells facilitate monocyte recruitment into the heart following MI via CCR2-MCP1 mediated trafficking and secrete high levels of pro-inflammatory mediators including IL-1β, TNF, and IL-6 (28). Not surprisingly, depletion of this cell population has resulted in reduced infarct size in a murine model of MI (28).

Conversely, CCR2^−^ macrophages appear to play an immune-modulatory, pro-regenerative role, expressing high levels of growth factors including Insulin-like growth factor 1 (IGF1) and Platelet derived growth factor C (PDGF-C) (28). Depletion of this CCR2^−^ population has enhanced monocyte/macrophage infiltration to the heart and further implicates these cells as potential immune-modulators during MI (28, 29). In addition to immune modulatory functions, recent studies have also demonstrated that CCR2^−^ macrophages express high levels of the sodium channel, SCN4, and sodium channel modulator, FGF13, suggesting that macrophages can modulate the electrical activity of cardiomyocytes (25, 30). Fracktalkine receptor (CX3CR1^+^) expressing resident macrophages have also been reported to facilitate conductivity, further implicating their role in regular functioning of the heart and broadening the role of macrophages beyond local inflammation and immune-modulation (30). It is not yet clear if both CCR2^+^ and CCR2^−^ macrophages contribute to the electrical homeostasis of the heart and, given that both subsets express the CX3CR1 (31), delineation of the direct impact of these individual cell-types on cellular conductivity is a promising avenue of exploration.

## The Extracellular Matrix

The ECM is a complex and dynamic structure of hundreds of numerous proteins which provide a support system for cells. Within the myocardium, it acts as a mechanical scaffold to create cellular and acellular networks. Conceptually, the cardiac ECM can be divided into two segments, the interstitial matrix, comprised of primarily type I and type III collagen, and the basement membrane, comprised of collagen IV, V, VII, X as well as laminins (32). Proteins residing in the interstitial matrix and basement membrane of the ECM serve a specific function, either facilitating structural support of the matrix itself, or regulating local cell behavior and function (33). Type I and Type III collagens for example allow the myocardium to maintain structural integrity through tensile support. Cardiac tissue undergoes mechanical stress via shear and pressure forces of muscle contraction and the organization and continuity of the collagen networks within the ECM allows for appropriate distribution of this physical stress. Elastin in the interstitial matrix provides elasticity to the cardiac tissue, with reduced expression post-MI contributing to stiffer scar tissue (34). Proteoglycans along with glycoproteins play a key role in signaling and turnover of the ECM (35), highlighting the alternative function of ECM as a transducer of signals within the cardiac environment.

The ECM is not an inert structure, with matrix continuously responding to signals from the surrounding environment and exerting its own signaling through mechanical and chemical cues. In the context of MI and chronic inflammation, disruption to the ECM via adverse remodeling leads to disarray of physical stress, applying strain on the myocardium and leading to dysfunction. Key initiators of remodeling can include ischemia, pressure overload, and aging of the heart, all of which have significant association with systemic inflammation (36–38) Thus, it is established that remodeling of the ECM as a consequence of sustained inflammation, is a critical etiological factor of heart failure, making the study of ECM and the innate immune activity in the failing myocardium one of great importance (39, 40).

## Role of Inflammation in Heart Failure -MI and Ischemia

**Figure 1:**
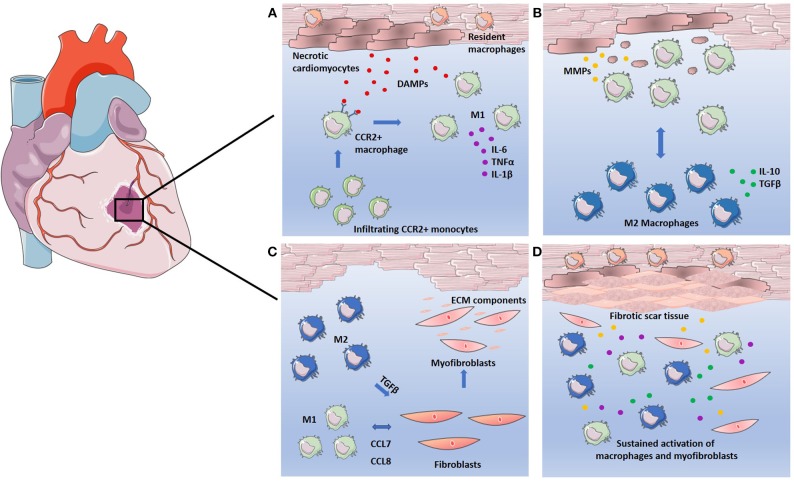
Macrophages in the response to infarction. **(A)** Cardiomyocytes undergo necrosis, releasing DAMPs and attracting CCR2^+^ circulating monocytes. CCR2^+^ monocytes differentiate into pro-inflammatory M1 macrophages replacing resident macrophages and secreting high levels of pro-inflammatory cytokines IL-6, TNFα, and IL-1β. **(B)** M1 macrophages clear necrotic cell debris through phagocytosis and induce breakdown of the ECM through secretion of MMPs. Phagocytosis of the necrotic debris causes macrophage polarization to the M2 phenotype. M2 macrophages secrete high levels of anti-inflammatory cytokine IL-10 and growth factor TGFβ. **(C)** Both M1 and M2 macrophages facilitate the fibrotic response. M1 macrophages recruit fibroblasts via CCL7 and CCL8 mediated signaling. M2 macrophages induce fibroblast differentiation into myofibroblasts, which in turn secrete ECM components to facilitate tissue repair. **(D)** Sustained activation of macrophages leads to continuous secretion of growth factors, pro-inflammatory cytokines, and MMPs. Continued breakdown of ECM as well as overproduction of ECM components by myofibroblasts leads to adverse remodeling of ECM and results in fibrotic scar tissue.

MI refers to mass cardiomyocyte death as a result of ischemia, which is often worsened by a subsequent reperfusion of oxygen supply, and the ensuing inflammatory response (41). This association between inflammation and adverse cardiac events is well-acknowledged. Multiple studies have demonstrated that elevated pro-inflammatory cytokine production in the heart correlates with worsening outcome. Furthermore, inhibition of pro-inflammatory cytokines, such as TNFα, which is heavily implicated in cardiac disease, results in improved cardiac function in rat models of heart failure (8, 9). Two classifications of infarction are often presented, both which occur as a result of ischemia. Acute MI is caused by an atherosclerotic plaque rupture causing coronary artery occlusion and cardiac tissue damage due to ischemia. Chronic MI refers to continued loss of cardiomyocytes from gradual and prolonged ischemia, often >8 weeks. The vast amount of cell death following MI poses a detriment, as the heart itself possesses a limited regenerative capacity (42). Left untreated, cardiac tissue undergoes extensive remodeling to compensate for cell loss and to maintain structural integrity. The inflammatory response facilitates the removal of necrotic cells in addition to tissue remodeling (43). However, extensive remodeling imposes stress on the heart, instigating maladaptive mechanisms, such as chronic inflammation and cellular apoptosis. As the heart continues to employ methods of remodeling and repair to resolve this, a destructive cycle prevails, ultimately leading to deterioration of cardiac function and heart failure (44). These events are summarized in Figure 1.

Infiltration of monocyte-derived macrophages to the infarct is a key feature of MI, and can be characterized by stages of macrophage infiltration, and their subsequent actions within the myocardium. Immediately following infarction, resident cardiac macrophages begin to die in response to ischemia, with a complete loss of resident macrophages within the infarct observed in murine models 24 h post-infarction (45). The resident macrophage population lost in the ischemic region is rapidly replaced by infiltrating monocyte-derived macrophages within 24 h (45). Day 1–3 post-MI, infiltrating macrophages exhibit a pro-inflammatory M1-like phenotype, driving acute inflammation and facilitating clearance of dead cells. At approximately day 5–7 post-MI, these macrophages begin to adopt a reparative M2-like phenotype, working to resolve inflammation and rebuild cardiac tissue (46). Mouse models of MI have revealed distinct subsets of infiltrating monocyte-derived macrophages, with earlier recruitment of pro-inflammatory Ly6C^high^ macrophages dependent on CCR2/CCL2 signaling, and the later pro-regenerative Ly6C^low^ macrophages recruited via CX3CR1 signaling (47). One can, therefore, hypothesize that inflammation and resolution is achieved in the myocardium through differential recruitment of macrophages.

It has also recently been demonstrated that infiltrating macrophages in murine mouse models of MI undergo metabolic reprogramming to increase oxidative phosphorylation at approximately day 5 post-MI (48). Increased oxidative phosphorylation in addition to fatty acid synthesis and oxidation is a signature of the M2 phenotype (49) and implies that there is a phenotypic switch from the early pro-inflammatory state to a more pro-regenerative one. Thus, not only is there the possibility of recruitment of separate subsets of macrophages via CCR2 and CXC3R dependent signaling, but in addition, there is a switch from the M1 to the M2f phenotype subset based on fatty acid synthesis and oxidation. This switch is key in the appropriate resolution of inflammation and progression to a pro-healing state and is promoted through efferocytosis of cell debris at the site of injury (50). Engulfment of dead cells by macrophages has been observed to elevate fatty acid synthesis and triggers production of the anti-inflammatory, pro-reparative cytokine TGFβ1 (20, 51). However, in circumstances of chronic ischemia or severe infarction, continuous cardiomyocyte death leads to sustained activation of M1 macrophages, which have a diminished efferocytotic ability (52). This is heightened in patients suffering from diabetes and obesity whereby underlying chronic inflammation exacerbates the pro-inflammatory response to infarction. In this instance, elevated levels of the pro-inflammatory cytokines, TNFα and IL-6, as well as C Reactive Protein (CRP), are associated with worse patient outcome (53, 54).

Failure to clear dying cells also leads to the release of damage-associated molecular patterns (DAMPs) which contribute to a robust secretion of pro-inflammatory cytokines, prolonging activation of the inflammatory response and escalating damage in the myocardium. A timely switch from low efferocytotic M1 macrophages to highly efferocytotic anti-inflammatory M2 macrophages is therefore necessary to clear dead cells and promote tissue repair.

## Alterations to the Extracellular Matrix

In any circumstances of injury within the body, ECM degradation is necessary to allow for repair and/or replacement of the damaged tissue. In the instance of MI, ECM degradation is triggered as early as 10 min following an ischemic event (55). MMPs, predominantly produced by macrophages and fibroblasts, are secreted as part of a programmed inflammatory response in order to deconstruct matrix architecture. As MI progresses, subsequent necrosis of cardiomyocytes accentuates matrix degradation. MMPs target various components of the ECM, for example, the collagenases MMP1, MMP8, and MMP13 cleave the α-chains of type I and type II collagens while MMP3 and MMP10 target proteoglycans, fibronectin, and laminins (33). Gelatinases, such as MMP2 along with MMP9 digest gelatin in addition to degrading type IV collagen, the most abundant component of the basement membrane (56).

As ECM breakdown persists, a provisional matrix enriched in fibrins forms in its place (57). This plasma-derived matrix does not serve a particularly structural role in the same manner as native ECM, but rather modulates cell phenotype and behavior, setting the stage for tissue repair. Components of this provisional plasma-derived matrix interact with migrating cells, such as macrophages via cell surface integrins. It has been hypothesized that the provisional matrix is capable of modulating gene expression and immune cell phenotype through these integrin-mediated interactions and, thus, progress the repair of cardiac tissue (58). Furthermore, the provisional matrix acts as a reservoir for numerous growth factors including PDGF, VEGF and members of the TGF family (59). These growth factors are secreted by pro-regenerative cells, such as M2 macrophages and subsequently deposited within the provisional matrix, bound via the heparin binding domain (59). Sequestering of growth factors in this fashion regulates their function and may influence the activation of fibroblasts and vascular cells. However, the full extent of these processes remains to be understood.

Healing of the infarct area greatly relies on clearance of this provisional matrix which has been clearly demonstrated in mouse models. In mice lacking the plasminogen/plasmin system responsible for this clearance, a lack of leukocyte infiltration into the infarct region is observed, and thus repair of the myocardium impeded (60). Fragments of the provisional matrix are endocytosed by CCR2^+^ macrophages and postulated to induce a switch to an anti-inflammatory, reparative state within the infarct area (61). *In vitro*, interactions with a fibrin matrix lead bone marrow derived macrophages to adopt an M2 like anti-inflammatory state (62), therefore it has been suggested that the fibrin enriched matrix and its removal promote an anti-inflammatory phenotype in local macrophages. Removal of the provisional matrix is followed by secretion of cellular fibronectin to form a cell-derived matrix (33). This matrix is enriched with macromolecules, such as thrombospondins, which facilitate the recruitment and activation of fibroblasts and macrophages to a pro-regenerative phenotype in order to promote healing.

The dynamics of the ECM, from native, to plasma derived, and then a cell-derived remodeled matrix, is a highly ordered process to allow for efficient transition from the inflammatory response to wound healing. Any anomalies in this process can lead to sustained inflammation and fibrosis, i.e., the accumulation of interstitial matrix components, predominantly collagen type I. Newly synthesized ECM differs from that of the original native ECM, with turnover of cross-linked collagen being significantly faster than that of normal collagen (63). This leads much stiffer collagen fibers, and ultimately, a stiff scar tissue post-MI. High expression of lysyl oxidase (indicative of cross-linking) has been observed in murine models of infarction, and correlates with a stiffer myocardium (64). In rat models of infarction, a 5-fold increase of lysyl oxidase is observed at day 3, and remains elevated at day 7 post-infarction (65). While the formation of scar tissue post-MI is important for maintaining structural integrity while the myocardium is under reconstruction, extensive scarring or remodeling limits the functional capacity of the heart by impeding ventricular contraction and relaxation (66). Furthermore, the detrimental effects of ECM remodeling extend beyond the infarct site as formation of scar peripheral to the site of infarction is also observed. This further limits myocardial function and heightens the progression of heart failure. To greater understand how these adverse effects arise, we look to the key mediators of ECM turnover.

## Macrophages as Mediators of ECM REMODELING

The cascade of events which lead to tissue remodeling post-infarction may be attributed to chronic inflammation and sustained activity of pro-inflammatory macrophages within the infarcted myocardium. In the early stages of infarction (Day 0–3), the mass influx of pro-inflammatory macrophages promotes clearance of matrix and debris through phagocytosis of dying cells and secretion of matrix proteases. The secretion of pro-inflammatory cytokines including TNFα and IL-6 by macrophages activates resident fibroblasts, which further increases the production of MMPS, such as MMP2 and MMP9 (67). These “immune-activated” fibroblasts also secrete pro-inflammatory cytokines including IL-1β and IL-6 in response to the macrophage secretome (68), which serves as a positive feedback and augments the pro-inflammatory response. While MMP production is required for natural matrix turnover, sustained activation of pro-inflammatory macrophages, and therefore continuous production of MMPs, results in extensive matrix remodeling and impaired wound healing. High levels of MMP9 have been reported in patients, serving as a biomarker for adverse left ventricle remodeling and heart failure (69). Mice overexpressing MMP14 also show lower survival and ejection fraction following MI (70). TIMPs can also contribute to adverse remodeling if produced in abundance as this can lead to unrestricted matrix deposition, thus highlighting the need for a controlled balance between MMPs and their inhibitors (71).

While the influx of pro-inflammatory macrophages enhances matrix breakdown post-MI, it is the transition from acute inflammation to fibrosis, facilitated by the switch from M1 to M2 dominant macrophage subsets that further exacerbates ECM remodeling. M2 macrophages secrete high levels of TGFβ1, which drives transcription of alpha smooth muscle actin (α-SMA) in the resident fibroblasts (72). As a result, these fibroblasts undergo a dramatic phenotypic transformation to become myofibroblasts (73, 74). Myofibroblasts have superior mobility compared to the homeostatic fibroblast and possess a higher capacity to produce matrix components (75). Macrophages not only amplify activation of these cells, but also facilitate their recruitment to the site of injury via signaling mediated by chemokines, such as CCL7 and CCL8 (76). As a result, overproduction of ECM components is observed, with an increased deposition of collagen, which stabilizes and crosslinks to form scar tissue. M2 macrophages can further promote fibrogenesis through the production of arginase, which activates glutamate and proline, both of which are necessary for collagen synthesis (77).

Clearly, both pro- and anti-inflammatory macrophages play a distinct pathological role in ECM remodeling, yet both subsets also have necessary roles in natural healing and repair. Therefore, it is difficult to pinpoint precisely which subset is a therapeutic target without further delineation of their functions in the infarcted myocardium. Certain pro-inflammatory cytokines, such as IL-6, elicit cardio-protective effects in the short term, and only pose a danger when their presence is sustained long-term (78–80). Furthermore, without CCR2^+^ pro-inflammatory macrophages, the clearance of fibrin-derived provisional matrix is impaired, thus limiting progression to the more permanent cell derived matrix (61). Moreover, a prolonged presence of fibrin fragments can prompt a pro-inflammatory response (58) and contribute to a state of chronic inflammation. Conversely, eliminating M2 macrophages can lead to a worsened outcome, as they are a potent source of IL-10. This anti-inflammatory cytokine exerts protection against cardiac fibrosis, with knockout murine models demonstrating that a lack of IL-10 leads to adverse tissue remodeling and more severe cardiac fibrosis when compared to wildtype counterparts (81). A more pragmatic approach, therefore, may be to harness the effects of the macrophages through immunomodulation rather than selective elimination.

## Immunomodulation: Targeted Therapy of Heart Failure

While clinical studies in MI patients are limited, strategies aimed at targeting dysregulated immune responses have been explored as treatment options for heart failure post-MI. Early trials involved the use of general immunosuppressants based on the hypothesis that non-specific inflammation following MI is unfavorable (82). Such trials included a broad range of candidates that are considered the gold standard of immunosuppression, such as corticosteroids, methotrexate and cyclosporin A to name but a few. However, their use in the context of cardiac treatment has yielded conflicting results. A review of clinical trials dating 1964 to 1989 reported that while corticosteroids appear to reduce mortality rates compared to placebo treatments, overall they do not provide a significant cardio-protective effect (82). Methotrexate, a well-established immunosuppressive routinely used for the treatment of rheumatoid arthritis, has also produced conflicting results with one trial reporting a reduction in TNFα and IL-6 together with an increase in IL-10 (83), while others reported no beneficial effects, instead worsening of left ventricle ejection fraction following treatment (84, 85). Cyclosporin A has also been considered for post-MI treatment given its ability to inhibit the mitochondrial permeability transition pore and therefore prevent necrotic cell death (86). However, no improvement of infarct size or mortality was observed in patients in a 3 day follow up (87).

While the lack of success for these drug trials may be attributed to short follow up periods and small sample numbers, it may also be that non-specific suppression of inflammation is insufficient to alleviate the adverse effects associated with infarction and more targeted approaches are required. IL-1β, for example, has proved to be a promising candidate to target due to elevated levels of the cytokine associated with poor prognosis in MI patients. Numerous pre-clinical studies have reported that inhibition of this cytokine results in reduced inflammation and remodeling in mice post-infarction (88, 89). Anakinra, an established antagonist for the IL-1β receptor; has been assessed in multiple pilot studies for efficacy in reducing left ventricular remodeling in patients (90–92). Antagonizing IL-1β in the above studies appears to blunt the acute inflammatory response exhibited post-infarction with an increase in pro-inflammatory marker CRP, observed following discontinuation of treatment. While the results of the 2010 study demonstrated an overall improvement in left ventricular remodeling, the small sample size proved limiting in their results. A larger study conducted in 2015 failed to show any improvement in remodeling compared to placebo treatment (91). Targeting IL-1β also reduces the risk of new MI events in patients with previous history of infarction. The CANTOS trial involving 10,061 patients, reported a 15% reduction in major adverse cardiovascular events upon treatment with the IL-1β targeting drug, canakinumab. However, no significant reduction in risk of cardiovascular death or overall mortality was observed (93).

Clinical trials targeting TNFα have also been conducted. In patients with acute MI, treatment with etanercept, a high affinity TNF receptor antagonist, resulted in reduced levels of IL-6 and lower neutrophil counts, however, no improvements in ventricular dilation or cardiac function were observed (94). Furthermore, in patients with chronic heart failure, trials involving anti-TNFα treatment were terminated prematurely due to lack of benefit (95). Despite encouraging preclinical results from *in vivo* models, targeting single cytokines alone may not be enough to counteract the complex pathophysiology associated with heart failure, and instead, targeting the source of inflammation may represent a more viable approach.

### Targeting Macrophages

Targeting macrophage infiltration to combat inflammation is not an entirely new concept, however many studies have failed to show a clear efficacy *in vivo*. Previous work using small molecule inhibitors to target the migration of CCR2^+^ macrophages, while showing a promising *in vitro* result, have failed to overcome challenges *in vivo* due to lack of tissue selectivity for the CCR2 receptor as well as poor potency in administered treatments (96). Advances in short interfering RNA (siRNA) technology, including improved specificity of targeting sequences, as well as new methods of delivery, have opened the door to novel therapies to treat inflammation and heart failure. For example, siRNA targeting of the cell adhesion molecules ICAM 1/2, VCAM, and E and P selectins have been shown to reduce inflammation and infarct size in a murine model of MI; ultimately preserving left ventricle ejection fraction and improving recovery after infarction (97). While this study emphasizes that a multi-targeted strategy may be necessary, targeting the CCR2 receptor alone has also yielded promising results with two separate studies demonstrating that siRNA-mediated targeting of the CCR2 receptor significantly reduces infarct size in mouse models (98, 99). Specifically, siRNA targeting the CCR2 receptor 1-h post-infarction (induced by coronary ligation) resulted in a 34% reduction in infarct size/area-at-risk at 24 h post-siRNA delivery (98). A similar study resulted in a reduction in expression of pro-inflammatory cytokines, IL-6, IL-1β, and TNFα at day 4 post-infarction, while levels of IL-10 appeared to increase (99). At 3 weeks post-infarction; a significant reduction of ventricular remodeling was observed compared to untreated mice (99). These results not only strongly implicate macrophages in the etiology of heart failure, but also demonstrate the ability to diminish their effects through single molecule targeting, which if tissue specific, may represent a viable option for future therapy. Targeting the CCR2 receptor proves particularly advantageous compared to pre-existing immunosuppressive treatments as the strategy does not affect the resident homeostatic macrophages present within the myocardium, nor does it hinder clearance of necrotic matter in the infarct. To improve siRNA delivery, nanoparticles and scaffolds are being extensively explored. Scaffolds can also be placed directly at the intended location of therapy. In particular scaffolds can also enable a controlled rate of delivery through interactions with the siRNA and designated target, as well as timed degradation of the scaffold itself (100, 101). This proves optimal for MI treatment whereby timing of inflammatory resolution is critical. Premature intervention of the inflammatory response may hinder wound repair, whereas a delayed response could fail to prevent adverse cardiac remodeling and heart failure (102).

### Targeting the ECM

Given that the ECM plays a pivotal role in driving macrophages activation, consideration for novel therapies should also be given to the interactions between cellular matrix and macrophage. For example, the role of the metalloproteinases is not limited to breakdown of ECM components; such enzymes also have a role in regulation of the inflammatory response through proteolytic cleavage of cytokines, chemokines, and growth factors (103). Protease mediated fragmentation of matrix proteins results in the generation of ECM-derived macromolecules known as matrikines which possess a distinct role in regulation of cell activity (104). Elastin fragments and collagen peptides are the most well-studied in this context and both have been implicated in numerous reports as activators of immune cells and fibroblasts during pro-inflammatory responses (105). Proline-glycine-proline, a tripeptide derived from collagen has been observed to act as a chemoattractant for neutrophil infiltration in models of pulmonary inflammation, in addition to promoting overall wound healing in mouse models. This peptide signals via the CCR2 chemokine receptor, which as mentioned previously, plays a prominent role in macrophage infiltration (106, 107). The dysregulated accumulation of these matrikines via continuous breakdown and remodeling of the ECM therefore may prove a detriment to the myocardium. Taking this into consideration, it is possible that the release of ECM fragments as well as their producers, the MMPs; hold promise as novel targets for the regulation of macrophage infiltration and subsequent inflammatory responses. Preliminary work to date has examined elastin and fibrillin-1 fragments which contain repeated Glycine proline motifs (GxxPG). In mice, neutralization of these GxxPG fragments via antibody administration reduces macrophage infiltration into the aorta as well as production of MMP2 and MMP9 (108). However, there have been little if any translational studies concerning the targeting of ECM fragments for cardiovascular treatment. This may be due to a substantial lack of knowledge surrounding the interactions between matrix fragments and the adverse inflammatory response within the myocardium.

**Figure 2:**
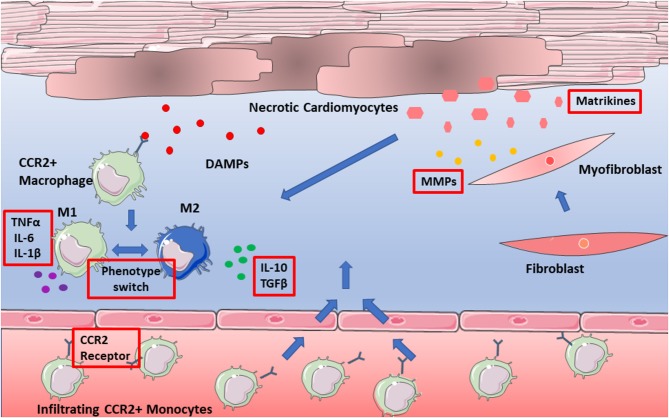
Potential therapeutic targets of adverse remodeling. Targets depicted in red boxes and include pro-inflammatory cytokines, as well as the CCR2 receptor. Also, to be considered are matrikines, which have yet to be assessed in targeted therapy of adverse remodeling.

Over recent years a plethora of matrikines have been recognized for their behavior-modulating abilities (summarized in Table 1), yet their precise mechanisms of action in sustaining inflammation remain to be elucidated. A focus therefore on interactions with ECM and the immune response, specifically with macrophages within the myocardium is required in future research. Breakdown of matrix will always be a natural requirement of wound healing and repair, yet perhaps it is the presence of matrikines or their dysregulation which contributes to adverse remodeling post-infarction. Their presence may have to be considered in future targeted therapy by means of combined therapy, where both effectors and actuators of remodeling are targeted. This is an important consideration going forward, in both therapeutic design, and research models of heart failure. Figure 2 depicts the numerous possible therapeutic targets of infarction and subsequent heart failure.

**Table 1:** ECM derived matrikines and their respective modulatory functions.

**Identified cryptid**	**Function**	**Source**	**References**
GETGPAGPAGPIGPVGARGPA, GPQGPRGDKGETGEQ	Facilitate wound healing via enhanced cell adhesion and antioxidative activities	Bovine collagen α-1(I) chain	(109)
RQVFQVAYIIIKA	Facilitate wound healing via enhanced cell migration	α-1 chain laminin	(110)
YGDEY	Antioxidant activity	Tilapia skin gelatin hydrolysates	(111)
KNVLVTLYERDEGNNLLTEK	Induces MMP9 production in monocytes	SPARC glycoprotein	(112)
VGVAPG	Induces MMP2 production in fibroblasts	Elastin	(113)
RGD	Cell adhesion via integrin binding	Fibronectin	(114)
DGGRYY	Activates polymorphonuclear neutrophils	A-1 chain type 1 collagen	(115)
GHK	Chemoattractant for macrophages and mast cells	A-2 chain type 1 collagen	(116)

## Concluding Remarks and Future Prospects

Undoubtedly, inflammation driven by macrophages plays a key role in heart failure. Numerous studies have been discussed in this review which pinpoint macrophages as critical mediators of inflammation and adverse remodeling of ECM. Yet there still remains substantial gaps in our knowledge of the precise role of macrophages, particularly resident macrophages within the myocardium. It remains to be established which specific subsets of macrophages are precisely responsible for the adverse effects of the inflammatory response, and which are necessary for normal homestatic function. Knockout models which eliminate specific subsets may bring to light the exact function of resident macrophages, and aid future research in harnessing their protective nature. As discussed throughout this review, although macrophages are not an active producer of ECM, they are intimately linked throughout the myocardial milleau in orchestrating remodeling and deposition; a role that becomes highly prominent following myocardial infarction. Greater efforts must be made to elucidate the role of the ECM in sustaining activation of macrophages via matrikines. Further studies of matrix-macrophage communication may reveal not only the precise mechanisms by which infiltrating macrophages drive remodeling, but also possible novel targets for future therapies.

## Author Contributions

MM, SO'R, and AD contributed to the conception and writing of the article. MM and SO'R wrote the first draft of the manuscript. All authors contributed to manuscript revision, read, and approved the submitted version.

### Conflict of Interest Statement

The authors declare that the research was conducted in the absence of any commercial or financial relationships that could be construed as a potential conflict of interest.
